# Progress in research on childhood T-cell acute lymphocytic leukemia, Notch1 signaling pathway, and its inhibitors: A review

**DOI:** 10.17305/bjbms.2020.4687

**Published:** 2021-04

**Authors:** Zhong Fang-Fang, Yang You, Liu Wen-Jun

**Affiliations:** 1 State Key Laboratory of Quality Research in Chinese Medicine, Macau University of Science and Technology, Macau, China; 2 Department of Pediatrics, Affiliated Hospital of Southwest Medical University, Birth Defects Clinical Medical Research Center of Sichuan Province, Luzhou, China

**Keywords:** Childhood leukemia, Notch1 signaling pathway, T-cell acute lymphocytic leukemia, T-ALL

## Abstract

Childhood leukemia is cancer that seriously threatens the life of children in China. Poor sensitivity to chemotherapy and susceptibility to drug resistance are the reasons for the treatment of T-cell acute lymphocytic leukemia (T-ALL) being extremely difficult. Moreover, traditional intensive chemotherapy regimens cause great damage to children. Therefore, it is highly important to search for targeted drugs and develop a precise individualized treatment for child patients. There are activating mutations in the *NOTCH1* gene in more than 50% of human T-ALLs and the Notch signaling pathway is involved in the pathogenesis of T-ALL. In this review, we summarize the progress in research on T-ALL and Notch1 signaling pathway inhibitors to provide a theoretical basis for the clinical treatment of T-ALL.

## INTRODUCTION

Leukemia is the most common cancer in children worldwide. It is estimated that there are approximately 15,000 new cases of childhood leukemia in China, more than 70% of which are accounted for by acute lymphocytic leukemia (ALL). T-cell ALL (T-ALL) accounts for 15% of childhood ALL, with the incidence rate being slightly higher in boys than in girls [1-3]. T-ALL is mainly manifested as diffuse bone marrow infiltration of immature T lymphoblasts, with early clinical manifestations such as central nervous system (CNS) infiltration and mediastinal mass accompanied by pleural effusion. Moreover, T-ALL has poor sensitivity to chemotherapeutic drugs and easily develops drug resistance. Furthermore, its recurrence rate is high in the CNS and it is difficult to induce remission and remove minimal residual lesions in the bone marrow. Therefore, the treatment for T-ALL is difficult, and the long-term prognosis is less than that of B-ALL [4-7]. With the current constant improvement in chemotherapy regimens there is a significant increase of 75% in the 5-year disease-free survival rate of child patients with T-ALL [8]. However, adverse reactions from intensive chemotherapy are also very obvious in child patients such as hepatic-renal dysfunction and severe infection secondary to bone marrow suppression that can threaten the lives of child patients. Therefore, it is pivotal to find targeted drugs for precise individualized treatment of child patients and overcome the adversity of large damage from the non-targeted treatment of traditional chemotherapy regimens to the normal body to increase the survival rate and reduce the recurrence rate of T-ALL.

An increasing number of scientists have investigated the Notch signaling pathway in the past century, initially discovering Notch mutants in *Drosophila melanogaster* [9,10]. Studies have demonstrated that the Notch signaling pathway involves the growth, development, proliferation, and apoptosis of T lymphocytes. A large number of in-depth genomics and molecular biological studies revealed that the abnormal activation of the Notch1 signaling pathway is closely related to the pathogenesis of T-ALL [11,12]. The Notch1 mutation in mammals was first observed in child patients with T-ALL. *NOTCH1* gene mutation of more than 50% has been found in child patients with T-ALL. The targeted therapy for this signaling pathway is currently a hotspot in research on T-ALL [13-17]. This study summarizes the progress in research on the T-ALL, Notch1 signaling pathway, and its inhibitors to provide a theoretical basis for the clinical therapeutic regimen of T-ALL.

## NOTCH1 SIGNALING PATHWAY

**FIGURE 1:**
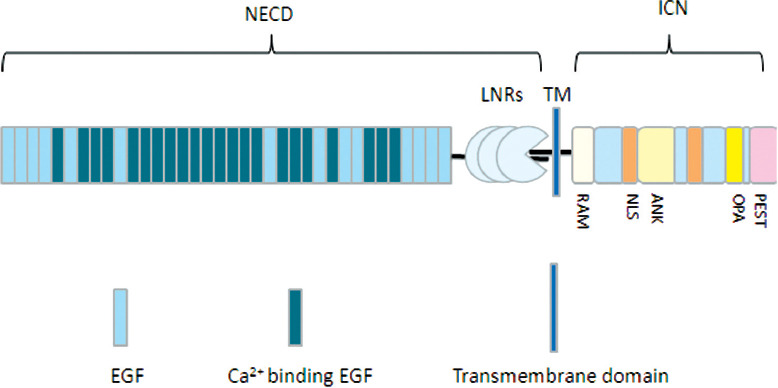
Modular organization of human Notch1. The Notch1 receptor is a heterodimeric type I transmembrane protein, including the extracellular domain, transmembrane domain, and intracellular domain. NECD: Notch extracellular domain; ICN: Intracellular domain of Notch1; LNRs: LIN-12/NOTCH repeats; RAM: Recombination binding-J-associated molecular; NLS: Nuclear localization signal; OPA: Glutamine-rich region; EGF: Epidermal growth factor.

Notch is a highly conserved transmembrane receptor protein family that widely exists in multiple species. Its signaling pathway plays an important role in the differentiation, proliferation, and apoptosis of normal tissue cells and pathological processes such as tumor and inflammation [18,19]. The Notch signaling pathway is composed of Notch receptor protein, Notch ligand protein, C-promoter binding factor 1 (CBF-1), suppressor of hairless (Su H), LIN-12 and GLP-1 (Lag-1) (CSL) DNA binding protein, and downstream target genes [20]. To date, four kinds of Notch receptor proteins have been found, namely, Notch1, Notch2, Notch3, and Notch4 and there are at least five kinds of Notch ligand proteins, namely, Jagged1, Jagged2, Delta-like 1 (DL1), DL2, and DL3 [21,22]. The Notch1 receptor is a heterodimeric type I transmembrane protein, which includes extracellular, transmembrane, and intracellular domains [8,23,24]. The extracellular domain is composed of multiple epidermal growth factor (EGF)-like repeats involved in receptor-ligand binding, 3 LIN-12/NOTCH repeats (LNRs) that connect 2 Notch subunits to stabilize the dimerization domain, and 1 heterodimerization domain (HD) that forms the Notch1 negative regulatory region (NRR) with LNRs, wherein NRR can make Notch1 dormant in the absence of ligand binding. The transmembrane domain contains two digestion sites (S2 and S3). The intracellular domain of Notch1 (ICN) or Notch intracellular domain consists of a series of cytoplasmic domains, including: 1) recombination binding-J-associated molecular (RAM) domain that can bind to CSL; 2) nuclear localization signal (NLS); 3) multiple ankyrin (ANK) repeats; 4) 1 glutamine-rich region (OPA); and 5) 1 C-terminal PEST domain that can regulate ICN degradation and renewal (Figure 1).

**FIGURE 2:**
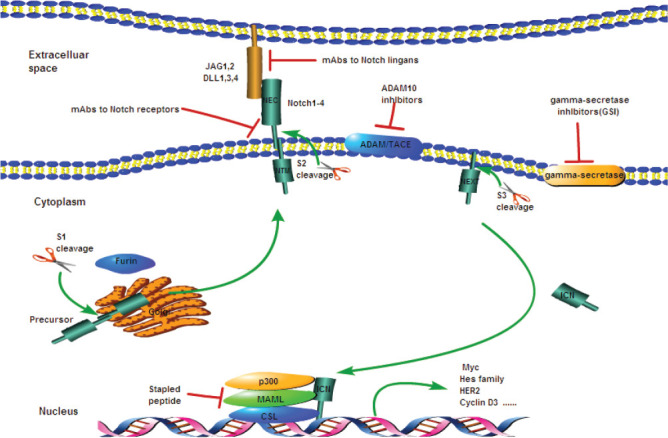
Notch1 signaling pathway and Notch1 inhibitors. GSIs and mAbs against Notch receptors or ligands are the two major classes of Notch inhibitors. ADAM: A disintegrin and metalloproteases; TACE: Tumor necrosis factor-α-converting enzyme; DLL: Delta-like ligand; JAG: Jagged; MAML: Mastermind-like; NEC: Notch extracellular subunit; NTM: Notch transmembrane fragment; NEXT: Notch extracellular truncated; CSL: CBF1/suppressor of hairless, and longevity-assurance gene-1; GSIs: γ-secretase inhibitors; mAbs: Monoclonal antibodies.

The Notch1 signaling pathway is activated after the Notch1 receptors bind to the ligands in adjacent cells. Studies have demonstrated that a longer time for enzyme digestion is required for its activation. The Notch1 precursor is cleaved by the furin-like convertase at S1 in the first enzyme digestion in the Golgi apparatus, forming two subunits that are connected into heterodimers by a non-covalent bond in the extracellular domain and transmembrane-intracellular domain. Then, the two subunits are transported and expressed as the transmembrane protein on the cell membrane, namely, the mature Notch1 receptor protein. The conformation of NRR will change when the ligands in adjacent cells bind to the extracellular domain of the mature Notch1 receptor protein, thus exposing the second digestion site (S2). Then Notch1 is hydrolyzed by a disintegrin and metalloproteases (ADAM10) into two fragments at S2, and the extracellular fragment is degraded, whereas the remaining fragment is hydrolyzed by γ-secretase at the third digestion site (S3), releasing the active fragment of Notch1, ICN1. ICN1 is rapidly transferred into the nucleus, and CSL DNA binding protein and mastermind-like (MAML) form the complex, promoting the target gene transcription of Hes1, c-Myc, interleukin 7 receptor α-chain (IL-7Rα), and insulin-like growth factor 1 receptor (IGF-1R). These key factors play important roles in the differentiation, proliferation, and apoptosis of a variety of cells, including T lymphocytes. Finally, the activated Notch1 is rapidly degraded in a targeted way by the F-box/WD repeat-containing protein 7 (FBXW7)–Skp1-Cullin-F-box (SCF) complex. FBXW7 is an E3 ubiquitin ligase that can identify the PEST of ICN1 and mediate the termination of Notch1 signal in the nucleus (Figure 2) [8,23,25,26].

## ABNORMAL ACTIVATION OF NOTCH1 SIGNALING PATHWAY AND T-ALL

**FIGURE 3:**
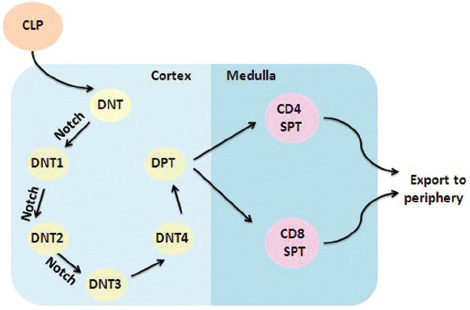
Notch in early T-cell development. The Notch signaling pathway plays an important role in the development of precursor T-cells into mature T-cells and the activation, proliferation, and differentiation of mature T-cells. CLP: Common lymphoid progenitor; DNT: Double-negative T-cell; DNT1: Double-negative T-cell 1; DNT2: Double-negative T-cell 2; DNT3: Double-negative T-cell 3; DNT4: Double-negative T-cell 4; DPT: Double-positive T-cell; CD4 SPT: CD4 single-positive T-cell; CD8 SPT: CD8 single-positive T-cell.

The Notch signaling pathway plays an important role in the development of precursor T-cells into mature T-cells and in their activation, proliferation, and differentiation (Figure 3) [27-29]. Multiple signaling pathways involved in T-ALL can be regulated through downstream target genes once the Notch1 signaling pathway is activated: 1) the expression of target gene *MYC* is regulated to activate the anabolism-related Notch1-MYC signaling pathway, promote the expression of anabolism-related genes, and further facilitate cell proliferation and metabolism [30,31]. 2) The G1/S differentiation of pre-T lymphocytes is promoted by upregulating the expression of cell division-related proteins, such as cyclin-dependent kinase 6 (CDK6) and G1/S-specific cyclin-D3 (CCDN3) [32,33]. 3) The downstream target gene *HES1* can upregulate phosphatidylinositol 3-hydroxy kinase (PI3K), thereby activating the PI3K-AKT-mTOR signaling pathway, which is an important pathway that regulates cell growth, proliferation, differentiation, and apoptosis and can antagonize the effect of the cancer suppressor gene, phosphatase, and tensin homolog deleted on chromosome 10 (PTEN) [34-36]. 4) The upregulation of downstream target gene NF-κB can activate the NF-κB signaling pathway and regulate the expression of the IL-7Rα chain, thus affecting the cancer suppressor gene *P53* and participating in cell differentiation and apoptosis [37,38]. Leading to massive uncontrolled growth and proliferation of nonfunctional pre-T lymphocytes and resulting in T-ALL, the constant activation of the Notch1 pathway significantly upregulates the key factors mediating the NF-κB signaling pathway that remains open. Demarest et al. [39] in their study implanted the *NOTCH1* gene mutation into hematopoietic stem cells of rats using a retrovirus, and T-ALL occurred in 100% of these rats. Some researchers also used zebrafish as experimental animals and demonstrated that the overexpression of *NOTCH1* gene due to its mutation will cause T-ALL [40]. It was also found in animal experiments that T-ALL is induced in 100% of mice implanted with ICN1 [41]. These studies have demonstrated that the abnormal activation of the signaling pathway caused by the activated mutation of *NOTCH1* gene in child patients with T-ALL is an important factor for T-ALL.

There are two types of mutations in the Notch1 signaling pathway in child patients with T-ALL, namely, Notch1 activated mutation and FBXW7 non-activated mutation. The Notch1 mutation mainly occurs in HD and the PEST domain [18,42] (Table 1). About 40% of the mutations occur in HD, dominated by HD1, HD2, and JME mutations [43,44]. The NRR composed of HD and LNR can conceal the digestion sites activated by the Notch1 signaling pathway, leading to loss of stability that can be broken in case of mutation in HD, continuously activating the pathway in the absence of ligand binding. In addition, HD mutation may affect the binding of the extracellular ligand binding and transmembrane domains of the Notch1 receptor, thus altering the conformation of heterodimer domain, making it easier for γ-secretase to digest and produce ICN, promoting further activation of the downstream signaling pathway, and upregulating transcription of target genes [45,46]. The mutations in the PEST domain are mostly nonsense mutations, which reduce the ICN ubiquitination and proteasome degradation, increase the amount of ICN in the nucleus, and lead to the continuous abnormal activation of downstream signals [24].

**TABLE 1:**
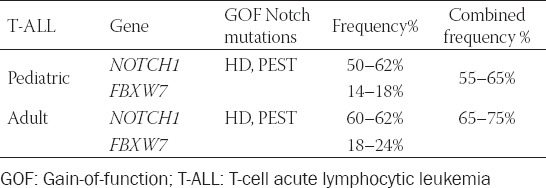
*NOTCH1* GOF mutations in T-ALL [47-52]

FBXW7 non-activated mutation occurs in more than 14% of child patients with T-ALL [47-52]. Such a mutation can weaken the ability of the FBXW7-SCF complex to identify the Notch1 active fragment ICN1 and impede the activated Notch1 to be degraded rapidly. At the same time, the mutated *FBXW7* gene can promote the protein expression of ICN1 so that the level of active Notch1 protein in the nucleus increases continuously, and the Notch1 pathway remains continuously activated, leading to T-ALL [53]. The Notch receptor and *FBXW7* mutations may simultaneously exist in patients (Table 1).

## NOTCH1 SIGNALING PATHWAY INHIBITORS

### γ-secretase inhibitors (GSIs)

GSIs were originally applied in the treatment of Alzheimer’s disease. The leading role of the Notch signaling pathway in the pathogenesis of T-ALL has been discovered, and γ-secretase has been revealed to be the third key enzyme in the activation process of the Notch signaling pathway. The inhibition on γ-secretase can block the Notch pathway, thus treating T-ALL caused by the abnormal activation of Notch1. A large number of cell and animal experiments were carried out using GSIs in resisting T-ALL, and currently, some promising treatment drugs have entered phase I clinical trial [54-56].

Using the mouse model of T-ALL caused by the *NOTCH1* gene mutation, Tatarek et al. [57] in this study found that GSIs can arrest the cell cycle in the G0/G1 phase, thereby inhibiting the abnormal proliferation and differentiation of T lymphocytes caused by the continuous activation of Notch1. At the same time, studies have also found that GSIs can promote apoptosis of T-ALL cell lines [58]. MK-0752 is a kind of noncompetitive oral GSI that has entered phase I clinical trial for the treatment of T-ALL. In the trial, seven T-ALL patients (*NOTCH1* gene mutation in four cases) took oral MK-0752 once a day for 28 days. The optimal therapeutic response was observed in one patient with activated mutation of Notch1, and the mediastinal mass was reduced by 45% at day 28, but there was no continuous remission due to severe gastrointestinal adverse reactions, and the disease developed at day 56 [58,59]. PF-03084014 is another GSI with clinical therapeutic potential. According to the latest phase I clinical trial, although the main adverse reactions of oral administration of PF-03084014 were nausea and vomiting, complete remission for 3 months was observed in one of eight T-ALL patients [60]. In phase I clinical trial of another GSI, BMS-906024, 25 child patients with recurrent/refractory T-ALL were treated with BMS-906024 with or without glucocorticoids; 32% of child patients who were treated with glucocorticoids had complete remission, one of which had no recurrence for 19 months [61].

Although GSIs have already entered clinical trial phase, the clinical response rate of GSIs, including the aforementioned three, is low or ineffective, and the reasons may be as follows: firstly, GSIs are a kind of pan-Notch inhibitors that also inhibit Notch1 and Notch2 signaling pathways in intestinal epithelial cells, for which the gastrointestinal adverse reactions are significant and the patients do not tolerate them, thus affecting the therapeutic effect. At the same time, GSIs can also promote the metaplasia of intestinal epithelial cells and differentiation of goblet cells, thus limiting its clinical application [62]. Secondly, there may be primary resistance in patients to GSIs, and GSIs directed at the Notch receptor protein may not be effective for T-ALL patients due to the inactivated mutation or deletion of PTEN and the mutation of FBXW7 [63-69].

Investigations pointed out a significant plateau in low and medium concentrations of GSIs. The therapeutic dose should be greatly increased to achieve an obvious therapeutic effect, which will also greatly increase the toxicity of GSIs and ultimately lead to intolerance of patients and clinical trial failure. In the face of this paradox, scientists are also working to reduce the toxic side effects of inhibitors and improve the lack of therapeutic response. Studies have found that the gastrointestinal effect of GSIs is based on a time- and dose-dependent manner; hence, the administration schedule is optimized and an intermittent administration is adopted to reduce the toxic side effects of GSIs without affecting efficacy [70]. The latest study also found that chloroquine (CQ), an antimalarial drug, can increase the therapeutic sensitivity of GSIs. This study showed that by adding CQ to γ-secretase inhibition, it causes a synergistic therapeutic effect on T-ALL and reduces the concentration of GSIs required to reduce cell viability and a block of proliferation [71]. Researchers have also investigated the application of GSIs combined with traditional chemotherapeutic drugs or other pathway inhibitors to improve its efficacy or produce a synergistic effect and reduce toxic side effects and drug resistance. Yoon et al. [72] combined vincristine (VCR) with GSIs and found that GSIs can remarkably enhance the efficacy of VCR. In some studies, GSI (PF-03084014) and glucocorticoids were jointly applied in glucocorticoid-resistant T-ALL patients, and it was found that the drug combination may upregulate the transcriptional expression of glucocorticoid receptor and glucocorticoid target genes and increase the drug sensitivity, thus exerting a synergistic antitumor effect. Meanwhile, in *in vivo* experiments, it has been found that, whereas hormones can alleviate the gastrointestinal adverse effects of GSIs, GSIs can improve glucocorticoid resistance in combined application [73,74]. Besides, GSIs are combined with other targeted therapeutic drugs that can cause T-ALL in a series of studies, such as PI3K-AKT-mTOR pathway inhibitors and NF-κB inhibitors, hoping to achieve a better therapeutic effect [75-81].

GSIs can inhibit cell cycle progression; hence, researchers have also analyzed the combination of GSIs with cell cycle inhibitors. Rao et al. [82] in their study applied GSIs combined with CCND1/CDK4 inhibitors and found that they can promote the apoptosis of T-ALL cells activated by Notch1. In another study by Pikman et al. [83], GSIs and CDK4/6 inhibitors were combined, and it was found that they can suppress the proliferation of T-ALL cell lines activated by Notch1. Unfortunately, however, no effect of the combined application on GSI-resistant T-ALL was found. Notch is involved in the body’s metabolic reaction; hence, researchers have also investigated the application of GSIs combined with metabolism and protein synthesis inhibitors [84]. Studies have found that bis-2-(5-phenylacetamido-1,2,4-thiadiazol-2-yl)ethyl sulfide (BPTES) can promote human T-ALL and PDX cells *in vitro* and *in vivo*; hence, researchers have also analyzed the combination of GSIs and BPTES to resist leukemia [84,85].

### Selective Notch inhibitors

Scientists are actively developing targeted therapeutic drugs with higher specificity as pan-Notch inhibitors have broader roles and more side effects. At present, the research hotspots of selective Notch inhibitors are the monoclonal antibodies (mAbs), which are directed at both Notch receptors and Notch ligands. Studies have demonstrated that mAbs selectively targeting the NRR of Notch1 receptors can inhibit and stabilize the NRR even in the case of HD1 mutation, with toxicity level lower than GSI. The combined application of mAbs and GSIs can also reduce the toxicity of GSIs, especially severe gastrointestinal adverse reactions [86]. The representative mAb is OMP-52M51 that can bind to the NRR of *NOTCH1* gene in a highly specific way, thus reducing HD mutation and effectively preventing the abnormal activation of ligand-independent signaling pathway caused by the HD mutation and the activation of ligand-driven Notch1 signaling pathway. As a result, the tumor cell growth is inhibited, and the survival time of mice is prolonged in animal experiments [87]. OMP-52M51 is currently in phase I clinical trial.

OMP-21M18 is a mAb against Notch signaling pathway ligand DLL4, which blocks the binding of the ligand to Notch1 and Notch4. OMP-21M18 is currently in phase Ib clinical trial, and it is able to significantly reduce the toxic side effects of GSIs [88,89]. However, mAbs can reduce the toxic side effects but lower the antitumor effect. The inhibitory effect of Notch mAbs is often less than that of GSIs in many *in vitro* experiments, including those in human T-ALL cell lines [86,90]. Of course, such drugs are mostly applied in clinical trials of solid tumors at present, and drugs suitable for blood tumors might be found by more research in the near future.

### Other inhibitors

In addition to GSIs, some pan-Notch inhibitors have also been in the development phase or in preclinical trials [91,92]. The mutation in HD accounts for 40% of the activated mutation of T-ALL *NOTCH1* gene [13], and such mutation reduces the stability of NRR, making the second digestion site S2 in hydrolysis of signaling pathway free from the control of NRR. Therefore, leading to the continuous abnormal activation of the downstream pathways, S2 is continuously exposed or exposed more easily and is continuously digested by ADMA10 [93]. Therefore, the occurrence of tumor can be prevented if ADMA10 can be selectively inhibited and its continuous digestion of S2 of mutant Notch1 can be blocked. In *in* *vitro* cell experiments, it has been found that the ADMA10 inhibitor GI254023X can inhibit the proliferation and induce the apoptosis of T-ALL Jurkat cell lines [94].

IGFR is a transmembrane tyrosine receptor and an expression product of the downstream target gene of Notch1. The PI3K-AKT-mTOR signaling pathway and membrane receptor tyrosine kinase signaling pathway initiate cell growth and development after IGF1 and IGF2 in cells binding to IGFR [95]. The abnormal activation of Notch1 significantly upregulates IGFR, leading to the long-term activation of signaling pathways and resulting in tumorigenesis [96]. In nude mouse experiments, Carboni et al. [97] found that the IGF-1R inhibitor BMS-536924 has an obvious inhibitory effect on Notch1 and PI3K-AKT-mTOR in T-ALL cells. BMS-536924 is currently in the preclinical trial.

Studies have also found that a polypeptide, SAHML1, is able to penetrate cells and target the formation of the ICN1-CSL-MAML1 transcriptional complex at the end of the Notch1 signaling pathway, thereby effectively blocking the Notch1 signaling pathway, and its potent antileukemia effect has been confirmed in the mouse model of T-ALL caused by *NOTCH1* gene mutation [98]. Similarly, in studies on MAML1 that acts on the transcriptional complex at the end of the pathway, and indicating that MAML1 may be a potential therapeutic target for T-ALL, the knockdown of the *MAML1* gene has been found to remarkably suppress the proliferation of T-ALL cell lines and induce the G0/G1 cell cycle arrest and apoptosis [99].

## CONCLUSION

The Notch1 signaling pathway plays an important role in the occurrence of T-ALL; hence, the targeted therapy using Notch1 pathway inhibitors has always been a research hotspot (Table 2). However, currently, in the clinical trial, GSIs and mAbs against the Notch1 receptor protein have such defects in treatment as low specificity and many adverse reactions, and they can distinguish between wild-type and mutant-type Notch1 [100]. Unfortunately, GSIs have shown limited clinical efficacy and dose-limiting toxicities. On the one hand, researchers are also developing treatment means that combine targeted inhibitors and traditional chemotherapeutic drugs, but on the other hand, they are improving the side effects of these drugs, reducing the side effects of inhibitors, and solving the problem of resistance to traditional chemotherapeutic drugs. Studies have also revealed that Notch1 has a synergistic effect with other oncogenes [101]; hence, the combined application with antitumor drugs may exert a more potent antitumor effect. At the same time, the more downstream the signaling pathway can be blocked by the drug, the fewer the side effects can be. Scientists are also investigating targeted inhibitors against the more downstream Notch1 pathway. At present, ADAM inhibitors and pathway target gene inhibitors are expected to become a new direction of research on targeted therapy to solve the dilemma of Notch1 pathway inhibitors and are in the *in vitro* study phase.

**TABLE 2:**
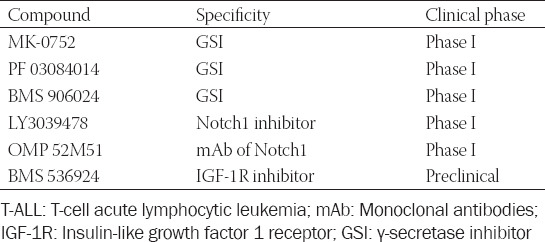
Promising targeted agents in Notch-dependent T-ALL
